# Enhancing routine HIV and STI testing among young men who have sex with men: primary outcomes of the get connected clinical randomized trial (ATN 139)

**DOI:** 10.1186/s12889-024-18522-w

**Published:** 2024-04-17

**Authors:** J.A. Bauermeister, K.J. Horvath, W.Y. Lin, J.M. Golinkoff, K.F. Claude, N. Dowshen, M. Castillo, P.S. Sullivan, M. Paul, L. Hightow-Weidman, R. Stephenson

**Affiliations:** 1 University of Pennsylvania, 418 Curie Blvd, Room 222L, 19104 Philadelphia, PA USA; 2 San Diego State University, San Diego, CA USA; 3 Florida State University, Tallahassee, FL USA; 4 Children’s Hospital of Philadelphia, Philadelphia, PA USA; 5 Emory University, Atlanta, GA USA; 6 Baylor College of Medicine, Houston, TX USA; 7 University of Michigan, Ann Arbor, MI USA

**Keywords:** mHealth, HIV prevention, Tailoring, Engagement

## Abstract

**Background:**

Regular HIV and STI testing remain a cornerstone of comprehensive sexual health care. In this study, we examine the efficacy of Get Connected, a WebApp that combines test locators with personalized educational resources, in motivating young men who have sex with men (YMSM) to undergo regular HIV and STI testing.

**Methods:**

Participants were randomly placed in one of two conditions. The first condition included the full version of GC (GC-PLUS), which included content tailored to users’ psychosocial characteristics (e.g., age, race/ethnicity, relationship status, HIV/STI testing history). The second condition served as our attention-control and only included the testing locator (GC-TLO) for HIV/STI testing services. Participants were recruited from three cities (Houston, Philadelphia, and Atlanta) characterized by high HIV incidence. Assessments were collected at 1, 3-, 6-, 9- and 12-month follow-ups.

**Results:**

Both versions of GC were acceptable and efficacious in increasing routine HIV and STI testing over a 12-month period. 40% of the sample reported testing at least twice, with no main effects observed across the two intervention arms (OR = 1.11; 95% CI: 0.69, 1.80), *p* =.66). Greater intervention effects were observed among YMSM who engaged more frequently with the intervention, with regional differences observed.

**Conclusions:**

Our findings underscore the need to cater to the diverse needs of YMSM through multilevel approaches. Broadly, mHealth HIV/STI testing interventions, such as Get Connected, would benefit from matching technologies to the local context to have the greatest impact.

**Trial Registration:**

This study is registered on ClinicalTrials.gov (NCT03132415).

Young men who have sex with men (YMSM) account for a significant proportion of new HIV and other sexually transmitted infections (STI) cases in the United States [1, 2]. HIV/STI testing services have the potential to reduce HIV and STI incidence by acting as a gateway to prevention (e.g., PrEP) or care (e.g., linkage to care) services [3]. Centers for Disease and Control (CDC) guidelines encourage individuals to test annually for HIV and other STIs, with populations at higher risk for HIV or STI acquisition (e.g., YMSM) being encouraged to test more frequently as needed [4]. Given their increased vulnerability to HIV/STI infections, YMSM are a group that may benefit from routine HIV/STI testing (i.e., at least two tests– 3 months apart - per year) [4].

Nationally representative data derived from 2006 thru 2019 of the National Survey of Family Growth found that 42% of YMSM (ages 15–24) reported ever testing for HIV and STIs [5, 6]. In non-representative samples of YMSM, researchers have found that between 25 and 60% of adolescent MSM (ages 13–18) have tested for HIV or STI in the past 12 months [7–10]. In studies with young adult MSM (ages 18–24), prevalence of testing in the prior year is around 40% for STIs [6] and 80% HIV [11]. However, less is known about the proportion of YMSM who engage in routine HIV or STI testing behaviors [12]. Routine HIV and/or STI testing estimates are less common in the literature, with estimates ranging from 20 to 37% in samples with YMSM [13–15]. Routine HIV and STI testing behaviors, however, requires that YMSM overcome a series of multilevel barriers [3, 16–24] across the individual (e.g., risk awareness, self-efficacy to get tested), systems (e.g., costs, medical mistrust, lack of culturally competent care), and structural (e.g., homelessness, stigma) levels. Therefore, developing strategies to promote HIV/STI status awareness among YMSM requires the creation of interventions that are responsive to the psychosocial needs of YMSM.

Online delivered HIV prevention interventions are ideal to reach and engage YMSM [25–29], and may be designed to circumvent some of the aforementioned challenges to routine HIV and STI testing [28–30]. Online interventions can deliver responsive and interactive content specific to each user’s characteristics (i.e., tailored content) and reach users across geographic regions [31]. Online content can also be refreshed to be contextually responsive over time, particularly as YMSM become sexually active, meet new partners and/or engage in different risk behaviors. While online interventions might offer tailoring at the individual level, recent interventions have also sought to extend tailoring to incorporate systems-level tailoring. In the Get Connected intervention [32], Bauermeister et al. incorporated youth-driven mystery shopper data [33] into the online intervention in order to match users with the most appropriate HIV/STI testing sites in their region through a tailored test locator feature. Preliminary efficacy results of the pilot randomized trial found that the fully tailored version of the intervention (i.e., GC-PLUS; systems-level tailoring through its test locator alongside individually tailored content to enhance HIV testing self-efficacy, resolve ambivalence about HIV prevention behaviors, and motivate testing) were more likely to report greater self-efficacy to ask partners to get tested for HIV/STIs (Cohen’s d scores ranged between 0.33 and 0.64), fewer sexual partners (Cohen’s d = 0.21), and increased HIV/STI testing behaviors (Cohen’s d = 0.34) at the 30-day follow-up when compared against a Get Connected attention-control version that only offered the systems-tailored test locator tool (i.e., GC-TLO). While both intervention conditions were deemed to be acceptable by YMSM in the pilot trial, it remains unclear whether the Get Connected intervention would be acceptable and efficacious across other regions in the United States. Moreover, given its design as a brief WebApp (i.e., a website that is optimized for smartphones and is like a mobile app in its appearance and functionality) intervention, it remains unclear whether the Get Connected intervention would encourage routine HIV/STI testing behaviors over a longer follow-up period. In the absence of these data, it is unclear whether the Get Connected intervention would be effective once scaled across other regions in the United States.

As part of the National Institutes of Health’s Adolescent Medicine Trials Network for HIV Interventions (ATN), we examined the acceptability and the efficacy of the Get Connected intervention [34] in promoting routine HIV/STI testing behaviors over a 12-month period among YMSM living in three metropolitan areas designated as Ending the HIV Epidemic [35] jurisdictions (Philadelphia, Atlanta, and Houston). Our study had three objectives. First, we examined the acceptability of both versions of the Get Connected intervention (i.e., GC-PLUS vs. GC-TLO). Consistent with the prior Get Connected pilot data with YMSM in Detroit [32, 36], we hypothesized that both intervention conditions would be acceptable among YMSM enrolled in the trial. Second, we examined the efficacy of the Get Connected intervention in promoting routine HIV and STI testing behaviors among YMSM living across the three regions. We hypothesized that the full version of Get Connected (GC-PLUS) would result in greater efficacy than the limited Get Connected version (GC-TLO) in promoting routine HIV and STI testing behaviors. Third, we examined whether there were differences in the intervention’s efficacy across regions given that the Get Connected intervention relies on local data to derive its systems-level tailoring. We hypothesized that there would be no differences between regions. Finally, we examined whether users’ intervention engagement moderated the relationship between the intervention versions and routine testing outcomes [37–39]. Consistent with prior research, we hypothesized that greater engagement would result in greater observed intervention effects.

## Methods

### Study recruitment

The Get Connected Trial (ATN 139) recruited participants using a variety of recruitment strategies, including advertising in social media platforms and dating apps, as well as in-person recruitment events in the community. Online advertisements linked to a web-based study screener survey. In person recruitment was coordinated by the respective study staff in Philadelphia, Atlanta, and Houston. In-person recruitment materials (e.g., palm cards; posters) displayed a QR code which took interested individuals to the study screener when scanned. The IRB of the University of North Carolina at Chapel Hill approved all study procedures detailed in this protocol (16-3183). We obtained a Certificate of Confidentiality from the National Institute of Child Health and Human Development. This study was registered on ClinicalTrials.gov (NCT03132415) on April 24, 2017. Details on the full protocol can be found elsewhere [34, 40].

### Eligibility & screener

To be eligible for the study, participants had to report being between the ages of 15–24 (inclusive), self-identify as a cisgender male (i.e., assigned sex at birth as male and self-identifies as male), reside in Philadelphia, Atlanta, or Houston, self-report as HIV-negative or unsure of their HIV status, read and speak English, and report having had consensual anal sex with a male partner in the prior 6 months.

### Study procedures

During the enrollment visit, study staff verified the participant’s eligibility and reviewed the informed consent with the participant. A waiver of parental consent was granted for participants under 18 to avoid selection biases operating by only recruiting minors whose parents were both aware of and comfortable with their child’s sexual orientation. Study staff used a random number generator to assign participants to the two intervention conditions. Block randomization was generated using a random number allocation sequence, stratifying by city to ensure balanced allocation within each region. Within each region, participants were randomized on a 1:1 basis to the GC-Plus or GC-TLO condition and blinded to their intervention assignment.

Consented participants completed the baseline survey as part of their enrollment visit. Once completed, study staff helped participants create an account on the Get Connected web app. Participants were also shown how to set up and navigate the WebApp on their personal devices. Participants had access to the web app for the 12-month duration of the study. At the conclusion of the enrollment visit, participants were reimbursed for their travel expenses to the ATN site (e.g., public transit reimbursement or parking voucher) and received a $50 Amazon e-gift card.

Data collection occurred between November 1, 2017 and December 15, 2021. Given timeline and budget considerations, study recruitment was staggered by site, with the Philadelphia ATN site launching first, followed by Atlanta and Houston. Each site was expected to recruit a total of *N* = 120 YMSM. Of 2,963 individuals who completed the study screener, a total of 948 (Philadelphia: *n* = 392; Atlanta: *n* = 396; Houston: *n* = 160) were eligible. Study staff then invited eligible individuals to attend an in-person study enrollment visit at their respective ATN sites. Of the 948 individuals who were eligible, 285 (30%) individuals attended a study enrollment visit at their respective site (Philadelphia: *n* = 119; Atlanta: *n* = 121; Houston: *n* = 45). The ATN Houston site was unable to complete its recruitment given COVID-19 pandemic disruptions.

Follow-up visits occurred virtually. Participants were emailed follow-up surveys at 1, 3, 6, 9, and 12 months after their enrollment visit. ATN site staff called and texted participants to remind them to complete their follow-up surveys. Participants had 30 days to complete the 1-month follow-up survey and 45 days to complete the 3-, 6-, 9-, and 12-month follow-up surveys. We used an increasing incentive structure throughout the trial. Amazon e-gift cards were used: $20 for the 1-month survey, $25 for the 3-month survey, and $30 for the 6-, 9-, 12-month surveys.

**Fig. 1:**
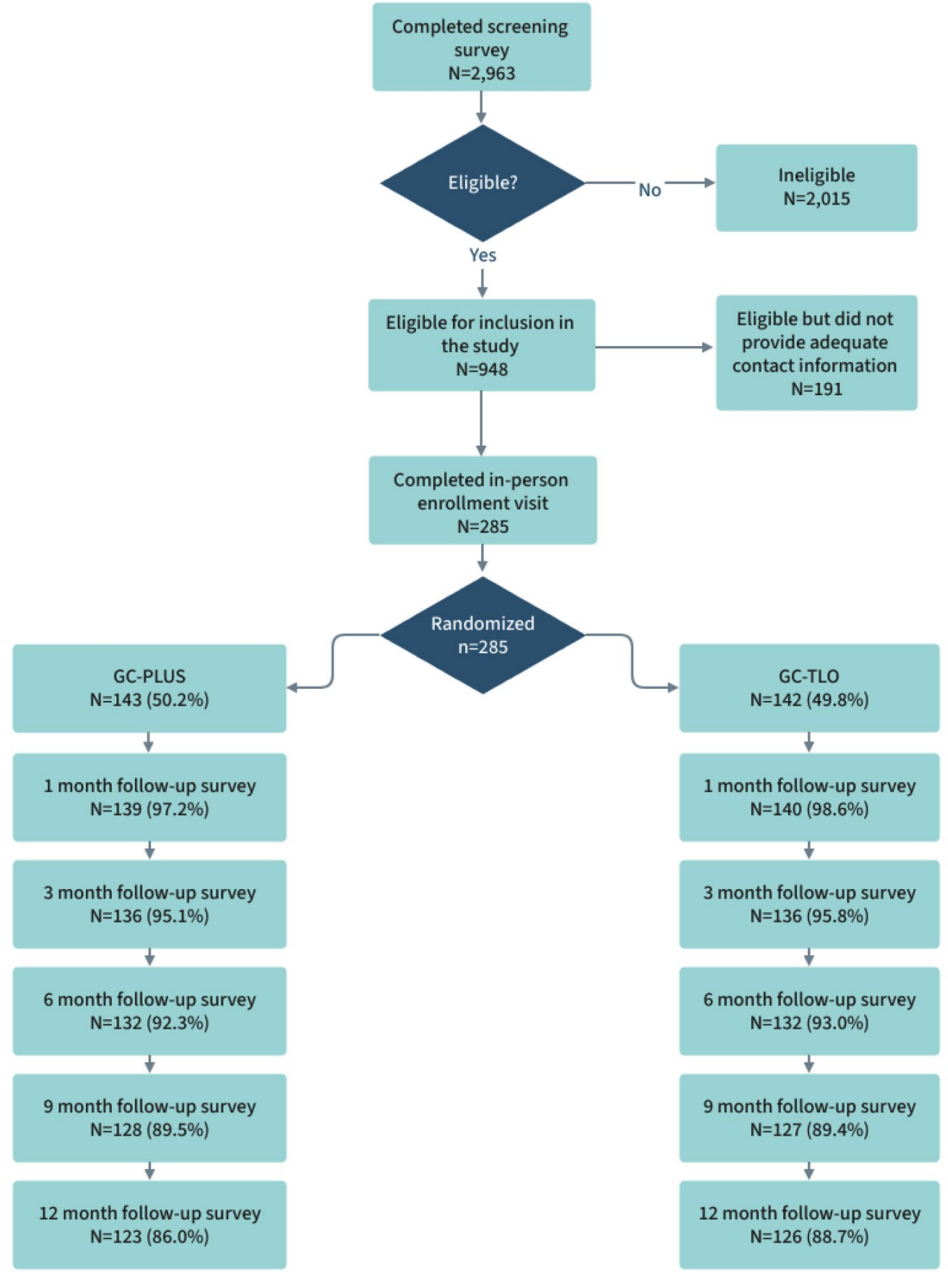
CONSORT Diagram

We present the study’s CONSORT diagram in Fig. 1. Overall, the participation rate was 96.5% across the 12-month period (i.e., 10 participants did not complete any follow-up surveys after baseline). 279 participants (97.9%) completed the 1-month follow-up survey, 272 participants (95.4%) completed the 3-month follow-up survey, 264 (92.6%) completed the 6-month follow-up survey, 255 (89.5%) completed the 9-month follow-up survey, and 249 (87.3%) completed the final 12-month survey. We found no differences in retention rates by study arm or recruitment city.

### Intervention description

**GC-PLUS**. In the intervention group, participants engage with a customized web application comprising four distinct sections labeled as “What,” “Why,” “How,” and “Where.” The “What” section, consisting of three pages titled “Facts,” “STIs,” and “Tests,” features topics presented in randomly organized boxes. These boxes open to reveal additional information upon user interaction. The Facts page imparts general prevention facts tailored to the specific population, such as “You won’t always know if someone has an STI.” On the STIs page, clicking on a specific STI, like “chlamydia,” provides users with detailed information on contraction, symptoms, testing options, and relevant treatments. The Tests page presents various HIV or STI testing methods, each box containing specific details accessible through user interaction.

The second section, focusing on the “Why,” consists of two pages: “Values” and “Pros and Cons,” following the design and functionality described in the “What” section. The Values page encourages participants to assess their motivations, values, and strengths related to HIV or STI testing. Tailored reasons for testing are based on participants’ testing history, acknowledging their prior behaviors. The Pros and Cons page presents information on the perceived benefits and barriers of testing.

The third section addresses the “How” of testing, featuring pages on potential “Barriers” and “Supports.” Barriers encompass issues like financial costs, social norms, and prioritization, impacting participants’ testing decisions. The “Supports” page provides information on how personal strengths and social support systems can aid in the decision-making process. Content on these pages is tailored to identify the most recent barriers and supports reported by YMSM in their recent survey.

The last section, the “Where” of testing, includes a page allowing users to “Customize” their search for nearby testing sites. A “Your Sites” page displays testing sites based on customization. Participants can customize their search based on clinic characteristics, such as walk-in availability, weekend hours, and insurance acceptance. Participants can email or text selected site information to themselves, along with seven questions developed by the GC youth and community advisory boards, proven helpful during pilot trials when interacting with test counselors perceived as ineffective.

Participants’ tailored content in each of these sections was derived from their baseline and follow-up survey data. Psychosocial indicators from these surveys were used to customize the content in our tailoring algorithms, including their race/ethnicity, sexual orientation, relationship status, HIV/STI testing history and testing motivations, recent sexual behavior, sources of support, structural barriers, and self-reported values. These data personalized both the images and text content. For example, a participant who had never tested for HIV received messages to promote the uptake of testing, whereas a participant with a prior HIV testing history received content that reinforced their prior testing behaviors.

Tailoring at the site level was informed by a mystery shopping method [40]. The intervention ranked providers in the “Your Sites” page based on baseline mystery shopper data. As participants visited sites to test for HIV/STIs over their 12-month study period, they were asked to complete the same mystery shopper indicators as part of their follow-up surveys. Participants’ scores were used to continually update the sites included in the intervention.

**GC-TLO**. Participants in the TLO version only had access to the “Where” test locator features of the GC-PLUS intervention.

### Measures

**Intervention Acceptability**: YMSM self-reported their acceptability of their assigned Get Connected condition at both the 3-month and 12-month follow-up surveys using the 10-item Systems Usability Scale [41] (SUS). The SUS is a validated scale answered on a 4-point scale (0 = Strongly Disagree; 4 = Strongly Agree) that ascertains two domains: participants’ perception of the information quality (5 items) and perceived usefulness of their intervention to improve their health (5 items). We derived a total score using the recommended SUS scoring (i.e., multiple each item by 2.5 and sum all items to create a Total SUS Score ranging from 0 to 100). Consistent with federal recommendations (https://www.usability.gov/how-to-and-tools/methods/system-usability-scale.html), we examined whether the two intervention conditions had a score of 68 or higher as it would indicate that the digital tool was “above average”. We also calculated the mean scores for the SUS subscales, where higher scores indicated endorsement that the intervention had greater information quality or perceived usefulness, respectively.

We created an overall mean satisfaction score [32] (α = 0.70) using three items assessed at the 12-month follow-up: “Overall, I am very satisfied with Get Connected”, “Using Get Connected is very frustrating” (reverse-coded), and “I would recommend Get Connected to my friends”. Each of these items could be answered using a 5-point scale (1 = Strongly Disagree; 5 = Strongly Agree). We also asked YMSM to report how likely they would be to continue using Get Connected if it were publicly available. Participants could answer this item using a 5-point scale (1 = Very Unlikely; 5 = Very Likely).

**HIV/STI Testing Behavior**. Our primary outcomes relate to the successful uptake of HIV/STI testing services. Our outcome was aligned with harmonized measures across ATN trials [42]. At baseline, participants were asked if they had ever tested for HIV and STIs, respectively. At each follow-up period, participants were asked to report if they had tested for HIV or STIs within the respective follow-up period window. The routine HIV testing outcome was defined as the proportion of YMSM who reporting testing for HIV at least twice − 3 months apart - during the 12-month follow-up period. We used the same operational definition for the routine STI testing outcome. If participants reported testing, they were asked to indicate whether a provider had diagnosed HIV or other STI.

**HIV and STI Risk Perception**. We asked participants to self-report their perceived risk for HIV (“Overall, how concerned are you about becoming HIV-infected?”) and STIs (“Overall, how concerned are you about getting a STI?”). These items could be answered from 0 = Not at all concerned to 10 = Extremely concerned. Risk perception was asked at both baseline and the 12-month follow-up survey.

**Intervention Engagement**. We measured intervention engagement [37] using the total number of logins from the intervention’s paradata. This engagement data was used to examine whether intervention dosage influenced the overall efficacy of the intervention.

**Sociodemographic characteristics.** Participants self-reported their age, race/ethnicity, sexual orientation, and relationship status. Participants also reported whether they lived alone, had been residential unstable in the prior 30 days (i.e., not having a consistent place to sleep), and had experienced food insecurity in the prior 3 months (i.e., having to skip a meal or cut a meal’s size because they did couldn’t afford its cost).

### Analytic plan

The primary outcome for the GC trial is HIV-negative and HIV-unknown YMSM’s successful uptake of routine HIV testing. The baseline HIV testing rate (in the previous 3 months) is expected to be 30–40%. A two-sided Z-test with pooled variance was used in calculating the sample size required for comparing the time-averaged difference between two proportions in uptake of routine HIV testing between GC-PLUS and GC-TLO from the baseline assessment to the 12-month follow-up assessment. Our estimates noted that sample sizes of 120 participants per intervention group would achieve 80% power to detect a difference of 0.10 in the uptake of routine HIV testing at an alpha level is 0.05.

We summarized the sociodemographic characteristics of our sample, overall and by ATN site (see Table 1). We used bivariate analyses to confirm that there were no differences in randomization. Differences in acceptability across the two versions of the Get Connected intervention (i.e., GC-TLO vs. GC-PLUS) were examined at the 3-month and 12-month follow-up assessments. This approach allowed us to see whether there had been any changes in YMSM’s perceptions of the intervention after having access to their version of the webapp across the 12-months.

**Table 1:** Sociodemographic characteristics of participants at baseline (*N* = 285), overall and by ATN site

	Overall M(SD)/*N*(%)	PHL (*n* = 119) M(SD)/*N*(%)	ATL (*n* = 121) M(SD)/*N*(%)	HOU (*n* = 45) M(SD)/*N*(%)	Test Statistic	*p*-value
Age	21.14 (2.09)	21.36 (1.95)	21.02 (2.12)	20.84 (2.33)	1.31	2.72
Race/Ethnicity					65.96	0.001
Non-Hispanic White Non-Hispanic Black Non-Hispanic Asian Latinx Multiracial Other	130 (45.6%) 52 (18.2%) 32 (11.2%) 57 (20.0%) 12 (4.2%) 2 (0.7%)	66 (50.8%) 18 (34.6%) 17 (14.3%) 10 (8.4%) 6 (5.0%) 2 (1.7%)	56 (46.3%) 29 (24.0%) 10 (8.3%) 20 (16.5%) 6 (5.0%) 0 (0%)	8 (55.5%) 5 (11.1%) 5 (11.1%) 27 (47.4%) 0 (0%) 0 (0%)		
Sexual Orientation					0.40	0.98
Gay Bisexual Other	211 (74.0%) 59 (20.7%) 15 (5.3%)	90 (75.6%) 23 (19.3%) 15 (5.3%)	88 (72.7%) 26 (21.5%) 7 (5.8%)	33 (75.6%) 10 (22.2%) 2 (4.4%)		
Single	138 (48.4%)	57 (47.9%)	57 (47.1%)	24 (53.3%)	0.53	0.77
Lives Alone	39 (13.7%)	17 (14.4%)	7 (15.6%)	15 (12.5%)	0.32	0.85
Food Insecurity	47 (16.7%)	22 (19.0%)	20 (16.5%)	5 (11.1%)	1.44	0.49
Residential Instability	15 (5.3%)	9 (7.6%)	5 (4.1%)	1 (2.2%)	2.41	0.30
Not tested for HIV (lifetime)	67 (23.5%)	25 (21.0%)	26 (21.5%)	16 (35.6%)	4.32	0.12
Not tested for STI (lifetime)	94 (33.0%)	34 (28.6%)	45 (37.2%)	15 (33.3%)	2.02	0.36
HIV Risk Perception	5.18 (3.06)	5.40 (2.68)	5.37 (3.16)	5.34 (3.12)	0.14	0.87
STI Risk Perception	5.33 (3.07)	5.51 (2.61)	5.21 (3.33)	5.29 (2.98)	0.17	0.84

Notes. PHL = Philadelphia; ATL = Atlanta; HOU = Houston

To examine the efficacy of the Get Connected intervention in promoting routine HIV and STI testing behaviors, we compared the proportion of YMSM who reported testing at least twice over the 12-month period by study arm. Given that the intervention relies on local data to derive its systems-level tailoring, we also examined whether the intervention’s efficacy was similar across the three ATN sites.

Finally, recognizing the role that user engagement may play in the evaluation of online HIV prevention trials, we examined whether engagement (i.e., total number of logins into the intervention) moderated the association between intervention assignment and routine HIV and STI testing behaviors, respectively. In exploratory analyses, we examined whether the association between YMSM’s engagement and their routine HIV and STI testing behaviors, respectively, varied by region and intervention arm.

## Results

A total of 285 YMSM between the ages of 15 and 24 who self-report as being HIV-negative or who are HIV serostatus unknown participated in the study. Consistent with our sampling design, over half of the sample was represented by racial/ethnic minority YMSM; however, ATN sites differed in their proportion of racial/ethnic sub-groups between their regions (see Table 1). Participants’ mean age was 21.14 years (SD = 2.09). Most participants identified as gay (*n* = 211; 74.0%), followed by bisexual (*n* = 59; 20.7%), or another sexual orientation (*n* = 15; 5.3%). A minority of participants reported living alone (13.7%), and 16.5% reported experiencing food insecurity. We observed no differences by intervention arm assignment or ATN site across these sociodemographic indicators.

Nearly a quarter of the sample (*n* = 67; 23.5%) had never tested for HIV at baseline. Among those who had ever tested, participants reported a median of three HIV tests, with nearly half having tested for HIV in the 3 months prior to baseline (*n* = 96; 44.0%). We observed no baseline differences on HIV testing history by intervention arm assignments (X^2^_(1)_ = 0.44, *p* =.51), or ATN site (X^2^_(2)_ = 4.32, *p* =.15). We observed no baseline differences across intervention arm assignments in participants’ perceived HIV (GC-PLUS: M = 5.01; SD = 2.95); GC-TLO: (M = 5.33; SD = 3.17); t(203) = 0.75; *p* =.45) or STI (GC-PLUS: M = 5.21; SD = 3.08); GC-TLO: (M = 5.45; SD = 3.08); t(256) = 0.62; *p* =.54) risk.

A third of participants (*n* = 94; 33%) had never tested for STIs before enrolling in the study. Of those who had ever tested for STIs in the past (*n* = 191), a third (*n* = 63; 33.0%) reported being diagnosed by a healthcare provider with an STI in the past. We observed no baseline differences in the percentage of participants reporting an STI by intervention arm assignment (X^2^_(1)_ = 0.04, *p* =.83) or ATN site (X^2^_(2)_ = 2.02, *p* =.36).

### Intervention acceptability

At the 3-month follow-up, both intervention versions had a SUS score above 68: GC-PLUS (M = 70.70; SD = 11.99) and GC-TLO (M = 72.14; SD = 12.83; t(258) = 0.93; *p* =.35). When we looked at subscales within SUS, we found that GC-PLUS (M = 3.31; SD = 0.53) was rated as having better information quality than GC-TLO (M = 3.19; SD = 0.66); t(265)=-1.68; *p* =.05; Cohen’s d = 0.21). YMSM on the GC-PLUS arm (M = 3.03; SD = 0.66) reported higher perceived usefulness of the intervention than peers assigned to the GC-TLO arm (M = 2.85; SD = 0.78); t(266)=-2.08; *p* =.02; Cohen’s d = 0.25).

Both intervention versions still retained comparable SUS scores at the 12-month follow-up (GC-PLUS: M = 72.50; SD = 13.43); GC-TLO: (M = 73.35; SD = 15.40); t(230) = 0.45; *p* =.66). GC-PLUS (M = 3.36; SD = 0.50) was perceived as having better information quality than GC-TLO (M = 3.17; SD = 0.66); t(236)=-2.39; *p* =.018; Cohen’s d = 0.31). At the 12-month follow-up, however, YMSM on the GC-PLUS arm (M = 2.98; SD = 0.75) reported comparable usefulness to peers in the GC-TLO arm (M = 2.85; SD = 0.80); t(235)=-1.29; *p* =.20). We observed no differences across SUS scores by ATN sites.

There were no differences at the 12-month follow-up in YMSM’s perceived satisfaction between the two arms (GC-PLUS (M = 3.99; SD = 0.69) vs. GC-TLO (M = 4.00; SD = 0.71); t(235) = 0.11;*p* =.91), or in their likelihood to use their assigned version of Get Connected were it publicly available in the future: GC-PLUS (M = 3.66; SD = 1.31) vs. GC-TLO (M = 3.64; SD = 1.29); t(235)=-0.07;*p* =.95). We observed no differences in satisfaction or future use across ATN sites.

### Efficacy outcomes

#### Routine HIV testing behaviors

Two thirds of the sample (*n* = 176; 64.0%) reported testing at least once over the 12-month period (GC-PLUS: *n* = 83; 60.1%; GC-TLO: *n* = 93; 67.9%; X^2^_(1)_ = 1.79, *p* =.19). 40% of the sample reported testing at least twice (see Table 2), with no statistically difference observed in the proportions across the two intervention arms (GC-PLUS: *n* = 60; 51.7%; GC-TLO: *n* = 56; 40.9%; OR = 1.11 (95% CI: 0.69, 1.80), *p* =.66). Two participants self-reported a new HIV diagnosis; both participants had been assigned to the GC-TLO version. We observed no differences in HIV risk perception by intervention arms at the 12-month assessment (GC-PLUS (M = 3.49; SD = 2.60) vs. GC-TLO (M = 4.09; SD = 2.77); t(184) = 1.53;*p* =.13).

**Table 2:** Routine HIV and STI testing outcomes among YMSM by intervention condition, overall and by ATN site

	Overall *N*(%)		OR (95% CI)	*p*-value	PHL *N*(%)		OR (95% CI)	*p*-value	ATL *N*(%)		OR (95% CI)	*p*-value	HOU *N*(%)		OR (95% CI)	*p*-value
	GC-PLUS	GC-TLO			GC-PLUS	GC-TLO			GC-PLUS	GC-TLO			GC-PLUS	GC-TLO		
Routine HIV Testing	60 (51.7%)	56 (40.9%)	1.11 (0.69, 1.80)	0.66	31 (52.5%)	17 (30.9%)	2.48 (1.15, 5.33)	0.02	19 (33.9%)	31 (50.8%)	0.50 (0.24, 1.05)	0.07	10 (43.5%)	8 (38.1%)	1.25 (0.37, 4.18)	0.72
Routine STI Testing	43 (31.2%)	46 (33.6%)	0.90 (0.54, 1.48)	0.67	24 (40.7%)	14 (25.5%)	2.01 (0.90, 4.46)	0.08	11 (19.6%)	25 (41.0%)	0.35 (0.15, 0.81)	0.01	8 (34.8%)	7 (33.3%)	1.07 (0.31, 3/78)	0.92

Notes. OR = Odds Ratio; CI = Confidence Interval; GC-PLUS: Full Intervention; GC-TLO = Test Locator Only Condition. For OR analyses, GC-TLO serves as the referent group

We also examined whether the intervention effects on routine HIV testing differed by ATN site (see Table 2). In Philadelphia, participants using the GC-PLUS version were more likely to engage in routine HIV testing behavior than peers using the GC-TLO version (53% vs. 31%; OR = 2.48 (95% CI: 1.15, 5.33), *p* =.02). We found no difference in routine HIV testing behaviors by intervention version in Atlanta (OR = 0.50 (95% CI: 0.24, 1.05), *p* =.07) or Houston (OR = 1.25 (95% CI: 0.37, 4.18), *p* =.72).

#### Routine STI testing behaviors

Half of the sample (*n* = 148; 53.8%) reported testing for STIs at least once over the 12-month period (GC-PLUS: *n* = 73; 52.9%; GC-TLO: *n* = 75; 54.7%; X^2^_(1)_ = 0.09, *p* =.76). A third of the sample (*n* = 89; 32.4%) reported testing at least twice for STIs, with comparable proportions across the two intervention arms (GC-PLUS: *n* = 43; 31.2%; GC-TLO: *n* = 46; 33.6%; OR = 0.90 (95% CI: 0.54, 1.48), *p* =.67). Among those who tested for STIs over the 12-month period, 41 reported a chlamydia diagnosis (GC-PLUS: *n* = 18; 36.0%; GC-TLO: *n* = 23; 52.3%; X^2^_(1)_ = 2.52, *p* =.11); 39 were diagnosed with gonorrhea (GC-PLUS: *n* = 23; 46.0%; GC-TLO: *n* = 16; 36.4%; X^2^_(1)_ = 0.90, *p* =.34); 21 had a syphilis diagnosis (GC-PLUS: *n* = 10; 20.0%; GC-TLO: *n* = 11; 25.0%; X^2^_(1)_ = 0.09, *p* =.76); 13 were diagnosed with herpes (GC-PLUS: *n* = 8; 16.0%; GC-TLO: *n* = 5; 11.4%; X^2^_(1)_ = 0.42, *p* =.52), and 4 received a genital warts diagnosis (GC-PLUS: *n* = 2; 4.0%; GC-TLO: *n* = 2; 4.5%; X^2^_(1)_ = 0.02, *p* =.90). One participant in the GC-TLO was diagnosed with Hepatitis B. We observed no differences in STI risk perception by intervention arms at the 12-month assessment (GC-PLUS (M = 3.90; SD = 2.91) vs. GC-TLO (M = 4.08; SD = 2.63); t(207) = 0.45;*p* =.65).

We observed differences in intervention effects when stratified by ATN site (see Table 2). In Atlanta, we found that routine STI testing was more frequent among YMSM in the GC-TLO condition than among peers in the GC-PLUS version (41% vs. 20%; OR = 0.35 (95% CI: 0.15, 0.81), *p* =.01). We observed no difference in routine STI testing between the two intervention versions in Philadelphia (OR = 2.01 (95% CI: 0.90, 4.46), *p* =.08) or in Houston (OR = 1.07 (95% CI: 0.31, 3.78), *p* =.92).

### Intervention engagement on routine testing

Participants reported an average of 6 logins (range: 1–41) over the trial period. We observed no differences between study arms (GC-PLUS: M = 6.65; SD = 5.29); GC-TLO: (M = 6.68; SD = 4.07); t(268) = 0.48; *p* =.97). In mean group comparisons by ATN sites (F(2,267) = 3.54; *p* =.03), YMSM enrolled in the ATN Houston site reported more logins (M = 8.36; SD = 6.46) than peers in the ATN Philadelphia (M = 6.36; SD = 4.77) and ATN Atlanta (M = 6.31; SD = 3.70). No statistically significant pairwise differences were found in the average number of logins between the Atlanta and Philadelphia sites.

#### Routine HIV Testing

When we examined whether YMSM’s total number of logins was associated with routine HIV testing (see Table 3), we found that number of logins was positively associated with routine HIV testing among YMSM assigned the GC-PLUS version (OR = 1.09 (95% CI: 1.01, 1.18), *p* =.003); however, we did not find an association between number of logins and routine HIV testing among YMSM in the GC-TLO version (OR = 0.97 (95% CI: 0.89, 1.06), *p* =.56).

**Table 3:** Routine HIV and STI testing outcomes among YMSM based on engagement with intervention conditions, overall and by ATN site

	Overall engagement OR (95% CI)				PHL OR (95% CI)				ATL OR (95% CI)				HOU OR (95% CI)			
	GC-PLUS	*p*-value	GC-TLO	*p*-value	GC-PLUS	*p*-value	GC-TLO	*p*-value	GC-PLUS	*p*-value	GC-TLO	*p*-value	GC-PLUS	*p*-value	GC-TLO	*p*-value
Routine HIV Testing	1.09 (1.01, 1.18)	0.003	0.97 (0.89, 1.06)	0.56	1.03 (0.93, 1.14)	0.58	0.97 (0.82, 1.15)	0.72	1.20 (1.02, 1.41)	0.03	0.97 (0.85, 1.11)	0.66	1.10 (0.91, 1.33)	0.31	0.94 (0.78, 1.15)	0.55
Routine STI Testing	1.11 (1.02, 1.21)	0.013	1.02 (0.94, 1.12)	0.63	1.07 (0.96, 1.19)	0.25	1.04 (0.87, 1.23)	0.67	1.27 (1.05, 1.54)	0.01	1.01 (0.88, 1.16)	0.88	1.08 (0.93, 1.24)	0.31	0.99 (0.82, 1.20)	0.92

Notes. OR = Odds Ratio; CI = Confidence Interval; GC-PLUS: Full Intervention; GC-TLO = Test Locator Only Condition

Recognizing that users may use the intervention differently based on the region in which they live, we examined whether the likelihood of routine HIV testing differed based on participants’ amount engagement with the intervention within each region. The number of logins among participants in Atlanta was associated with greater routine HIV testing in the GC-PLUS version (OR = 1.20 (95% CI: 1.02, 1.41), *p* =.03), but not in the GC-TLO version (OR = 0.97 (95% CI: 0.85, 1.11), *p* =.66). We found no evidence to suggest that total number of logins moderated the relationships between YMSM’s assigned intervention version and routine HIV testing in Philadelphia (GC-PLUS: 1.03 (95% CI: 0.93, 1.14), *p* =.58; GC-TLO: 0.97 (95% CI: 0.82, 1.15), *p* =.72) or in Houston (GC-PLUS: OR = 1.10 (95% CI: 0.91, 1.33), *p* =.31; GC-TLO: OR = 0.94 (95% CI: 0.78, 1.15), *p* =.55).

#### Routine STI testing

Overall, the number of logins was associated with routine STI testing among YMSM assigned the GC-PLUS version (OR = 1.11 (95% CI: 1.02, 1.21), *p* =.013); but not in the GC-TLO version (OR = 1.02 (95% CI: 0.94, 1.12), *p* =.63).

When we examined the associations between intervention engagement and routine STI testing by ATN sites, we found that the total number of logins moderated the relationship between routine STI testing and the intervention version among YMSM in Atlanta. Specifically, we found participants in Atlanta who were assigned to the GC-PLUS version were more likely to engage in routine STI testing if they had logged onto the site more often (OR = 1.27 (95% CI: 1.05, 1.54), *p* =.01); however, we did not see an association between number of logins and routine STI testing among participants in the GC-TLO version (OR = 1.01 (95% CI: 0.88, 1.16), *p* =.88). The total number of logins did not moderate the relationships between routine STI testing and intervention version among YMSM in Philadelphia (GC-PLUS: 1.07 (95% CI: 0.96, 1.19), *p* =.25; GC-TLO: 1.04 (95% CI: 0.87, 1.23), *p* =.67) or in Houston (GC-PLUS: OR = 1.08 (95% CI: 0.93, 1.24), *p* =.31; GC-TLO: OR = 0.99 (95% CI: 0.82, 1.20), *p* =.92).

## Discussion

This study provides valuable insights into the acceptability and efficacy of two versions of the Get Connected intervention among YMSM regarding HIV and STI testing behaviors. Our study revealed that a sizable proportion of YMSM had never tested for HIV or STIs at baseline, indicating the need for interventions targeting this high-risk group. Notably, there were no differences in testing history or risk perception among the two intervention arms, ensuring a comparable starting point for both groups.

Both versions of Get Connected were deemed acceptable by YMSM. Importantly, both GC-PLUS and GC-TLO interventions maintained comparable SUS scores over a 12-month period, indicating sustained usability and user-friendliness over time. This stability in SUS scores for the two intervention arms across time suggests the robustness of the overall user experience. Furthermore, YMSM participants reported comparable satisfaction levels regardless of the specific intervention version they were assigned. While both versions maintained comparable overall acceptability scores, nuanced differences emerged. The GC-PLUS version was perceived to have better information quality and higher perceived usefulness, suggesting that certain aspects of the intervention might be more beneficial for participants. This difference is consistent with the design of the GC-PLUS version, as only YMSM in this version of the intervention received additional individual-level tailored HIV/STI prevention content. However, these differences leveled out over time, indicating a stabilization of perceived usefulness between the two versions by the 12-month follow-up. Taken together, these findings suggest that both intervention versions could be scaled for widespread adoption without interfering with its overall acceptability.

Our results indicate that the Get Connected intervention encouraged routine HIV/STI testing behaviors and supports earlier pilot work [32]. This may be attributed to the tailored nature of our intervention, which addressed multilevel barriers and concerns that traditionally hinder YMSM individuals from testing routinely [43]. The observed increase in testing frequency is promising, as it signifies a positive impact on the participants’ awareness and proactive engagement in their sexual health. The higher testing rates in both intervention versions, as compared to the previously reported estimates, demonstrate the efficacy of our approach in encouraging routine HIV and STI testing among YMSM. We acknowledge, however, that the absence of a no-treatment control group limits our ability to test the superiority of either version of Get Connected in promoting routine HIV and STI testing behaviors. Although this approach would have been preferable to benchmarking our intervention’s outcomes to the published literature, it felt unethical to withhold HIV prevention resources to YMSM across the three regions given their vulnerability to HIV and STIs.

We also noted an overall positive association between participants’ engagement (i.e., number of logins) and routine HIV and STI testing over time across both versions of the intervention. This finding is consistent with prior research noting the importance of paradata in online interventions and suggests that users’ engagement with a digital intervention may result in differential efficacy [37, 39, 44, 45]. In other words, participants may yield greater benefits from a digital intervention if they engage with it more often. Regional differences can also influence individuals’ access to HIV prevention resources and other healthcare systems [35]. This highlights the importance of considering regional variations in the efficacy of online interventions. Taken together, these findings emphasize the need to deepen our understanding of how online delivered interventions may result in differential efficacy and effectiveness based on geographical differences (e.g., availability and access to HIV and STI testing settings). For example, during the mystery shopping component of this trial [40], we observed regional differences in the number of youth-friendly HIV/STI testing sites in each region (e.g., 38 of 53 agencies (57%) in Philadelphia sites offered free, walk-in HIV testing using a rapid test, as compared to 19 of 46 agencies (41%) in Houston or 17 of 50 agencies (34%) in Atlanta). Similarly, we observed differences in YMSM’s perceptions of LGBTQ + visibility between regions: Atlanta sites had greater materials indicative of LGBTQ + inclusiveness than the Philadelphia or Houston sites, respectively. By combining both systems-level tailoring with individual-level tailored content, our intervention acknowledges the heterogeneity within the YMSM community, recognizing that individual needs may vary across regions. Taken together, this tailored approach moves away from region-agnostic, online interventions towards online tools that situate users within their given social environment. In other words, online interventions cannot be designed as a “one size fits all” when it comes to regional variations, as users’ social contexts may facilitate or deter their ability to engage in the behaviors being proposed through the online intervention.

While one of the key strengths of Get Connected is its ability to provide individual-level and/or systems-level tailored HIV prevention content, the observed effects of the intervention may vary due to a combination of geographic location and user engagement. For instance, when we analyze the overall effects without considering location or engagement, we find similar rates of HIV and STI routine testing across both versions of Get Connected. However, upon further investigation by location, we discover a different outcome: YMSM in Atlanta using the GC-TLO version are more likely to report routine STI testing compared to those using the GC-PLUS version. Since user engagement can impact how the intervention affects testing outcomes, we then examined if there is a relationship between engagement levels and testing frequency in these location-based analyses. In line with our overall findings on engagement, we observed that higher engagement increases the likelihood of routine STI testing among users of the GC-PLUS intervention. However, we did not find a clear dose-response relationship among users of the GC-TLO version. Additionally, we did not find similar dose-response patterns in Philadelphia or Houston. In summary, our findings suggest that when evaluating the effectiveness of digital interventions, it’s crucial to consider both the geographical distribution of users and their level of engagement with the intervention. Future research examining the interplay between geospatial characteristics and users’ engagement when evaluating the effectiveness of online interventions may be warranted.

It is important to acknowledge the limitations of our study. First, the COVID-19 pandemic hindered our ability to complete the recruitment of YMSM in the Houston site. As a result, our ability to make inferences about the intervention’s efficacy in Houston is limited by the small sample size enrolled prior to the pandemic. Second, socioecological confounders might explain the observed differences by region. Our stratified analyses by ATN region should be interpreted with caution. Future research examining to what extent regional characteristics (e.g., population density; overall size of regions; HIV/STI prevention infrastructure and policies; access to public transportation) may influence HIV/STI testing is warranted. Moreover, future implementation science research may be warranted as the intervention is scaled and sustained with larger sample sizes in order to examine these regional variations with greater precision. Third, although we had high response rates across the 12-month period, we were unable to confirm participants’ testing behaviors or self-reported HIV and STI diagnoses. However, the self-reported prevalence of new HIV and STI cases aligns with the known need to reduce HIV/STI incidence in this population. Finally, given that participants report their testing behaviors at each follow-up, it is possible that survey questions might have unintentionally reinforced HIV/STI testing behaviors and increased participants’ social desirability.

Despite these limitations, our findings underscore the value of how tailored interventions can indeed lead to improvements in routine HIV and STI testing behaviors among YMSM. Our study demonstrates the overall acceptability of both intervention versions of Get Connected among YMSM. The findings from this 12-month follow-up evaluation highlight the enduring usability, perceived information quality, and user satisfaction. Moreover, our results underscore the value of both versions of the Get Connected intervention and provide guidance for the development and implementation of future multilevel, tailored interventions online. Tailoring interventions to regional and individual needs, coupled with a focus on enhancing user engagement, could be key to maximizing the impact of interventions aimed at promoting HIV and STI testing among YMSM. By acknowledging and addressing these regional differences, online HIV prevention interventions can be customized to meet the specific needs of diverse communities, ensuring a more effective and inclusive approach to HIV prevention.

## Data Availability

The datasets generated and/or analyzed during the current study are available in the Eunice Kennedy Shriver National Institute of Child Health and Human Development (NICHD) repository, under the Data and Specimen Hub (DASH): https://dash.nichd.nih.gov/.

## Abbreviations

ATN: Adolescent Medicine Trials Network for HIV/AIDS Interventions
CDC: Centers for Disease Control and Prevention
CI: Confidence Intervals
ES: Effect sizes
GC: Get Connected
IRB: Institutional Review Board
LGBTQ: Lesbian, gay, bisexual, transgender, questioning or queer
M: Mean
MSM: Men who have sex with men
NIH: National Institutes of Health
OR: Odds Ratio
PrEP: Pre-exposure prophylaxis
RCT: Randomized controlled trial
SD: Standard Deviation
SUS: Systems Usability Scale
STI: Sexually transmitted infections
YMSM: Young men who have sex with men
